# Identification of Hub Genes and Immune Infiltration in Non-alcoholic Fatty Liver Disease -Related Hepatocellular Carcinoma by Bioinformatics Analysis

**DOI:** 10.5152/tjg.2023.22590

**Published:** 2023-04-01

**Authors:** Xu Liu, Yan Wang, Tao Li, Yundong Qu

**Affiliations:** 1 Department of Infectious Diseases and Hepatology, The Second Hospital of Shandong University, Jinan, Shandong, China

**Keywords:** Non-alcoholic fatty liver disease, hepatocellular carcinoma, bioinformatics analysis, hub genes

## Abstract

**Background:**

Non-alcoholic fatty liver disease has been a significant risk factor for hepatocellular carcinoma. In the study, we aimed to identify the key genes associated with the transition from non-alcoholic fatty liver disease to hepatocellular carcinoma through bioinformatics analysis.

**Methods:**

The GSE164760 dataset was used for identifying differentially expressed genes. Gene Ontology and Kyoto Encyclopedia of Genes and Genomes enrichment analysis were performed to explore the potential function of the differentially expressed genes. Subsequently, the protein–protein interaction network was constructed to select hub genes, and the immune cell infiltration was analyzed. Finally, the receiver operating characteristic analysis was performed to assess the diagnostic ability of the crucial genes.

**Results:**

A total of 156 differentially expressed genes were identified. Gene Ontology enrichment analysis indicated that differentially expressed genes were strongly associated with cellular hormone metabolic process, response to xenobiotic stimulus, collagen-containing extracellular matrix, detoxification, and regulation of growth. In the protein–protein interaction network, *ESR1*, *CAT*, *CXCL8*, *CD4*, *SPP1*, *CYP2E1*, *CYP3A4*, *UGT2B7*, *GSTA1* and *THBS1* were selected as the hub genes. Immune infiltration analysis demonstrated that M0 macrophages, plasma cells, CD8+T cell and M2 macrophages were significantly changed in tumor tissues. Finally, we verified the hub gene expression and selected *CD4*, *UGT2B7*, and *CYP3A4* as the potential diagnostic biomarkers.

**Conclusion:**

*CD4*, *UGT2B7*, and *CYP3A4* were selected as the potential diagnostic biomarkers of non-alcoholic fatty liver disease–hepatocellular carcinoma.

**Main Points**

- Non-alcoholic fatty liver disease (NAFLD) has become an important risk factor for hepatocellular carcinoma (HCC).
- M0 macrophages, plasma cells, CD8+T cells, and M2 macrophages were significantly changed in NAFLD-HCC tissues.
- *CD4,* *UGT2B7*, and *CYP3A4* were selected as the potential diagnostic biomarkers of NAFLD-HCC.

## INTRODUCTION

Non-alcoholic fatty liver disease (NAFLD) comprises a spectrum of diseases varying from liver steatosis to non-alcoholic steatohepatitis (NASH); the latter can progress to cirrhosis and become an emerging risk factor for hepatocellular carcinoma (HCC).^1-3^ Although the current incidence of NAFLD-associated HCC is still lower than other etiologies such as hepatitis B, its prevalence is expected to increase with the obesity and metabolic syndrome epidemic.^4^ Therefore, NAFLD-induced HCC is entirely worthy of clinical attention.

Non-alcoholic fatty liver disease-associated HCC, which often occurs in the absence of cirrhosis, is less likely to be detected than HCC arising from other etiologies.^5,6^ The majority of patients with NAFLD-HCC are diagnosed at advanced unresectable stage requiring systemic treatment. Currently, immunotherapy has been approved for NAFLD-HCC. However, emerging evidence indicates that HCC arising from NAFLD might be less responsive to immunotherapy.^7,8^ Both tumor and tumor immune microenvironment (TME) factors determine the poor response.^9^ Therefore, more studies are needed to explore the mechanism and identify more effective diagnostic biomarkers for NAFLD-HCC.

In this study, we downloaded the NASH-HCC microarray gene expression profile (GSE164760) from the Gene Expression Omnibus (GEO) database for bioinformatics analysis. By comparing the gene expression between NASH tissues and NASH-HCC tissues, we screened out differentially expressed genes (DEGs) and constructed protein–protein interaction (PPI) network for selecting hub genes. Furthermore, we performed the immune cell infiltration analysis and verified the hub genes in another dataset (GSE164441). Finally, we discovered that *CD4*, *UGT2B7*, and *CYP3A4* may have the potential to serve as diagnostic biomarkers in the progression of NASH to HCC. The results may provide novel biomarkers for NAFLD-related HCC diagnosis and facilitate the development of targeted therapeutics.

## MATERIALS AND METHODS

### Data Collection and Preprocessing

The datasets for NAFLD-HCC (GSE164760^10^ and GSE164441^11^) were downloaded from the GEO (https:// www.ncbi.nlm.nih.gov/geo/) database by R version 4.2.0 using the “GEOquery” R package. The dataset GSE164760 includes 53 NASH-HCC samples and 74 NASH samples. The dataset GSE164441 contains 10 paired NAFLD-HCC tissues and adjacent non-tumor liver tissues. We filtered out probes without corresponding gene symbols. For multiple probes annotated to the same gene, we retained the gene randomly.

### Differentially Expressed Gene Screening

The “limma” R package was used to select DEGs between NASH-HCC samples and NASH samples, as previously described. Threshold values were set as *P* <.01 and |log_2_FoldChange|>1. The “ggplot2” and “pheatmap” packages were applied to visualize the significant DEGs.

### Functional and Pathway Enrichment Analysis

Metascape (http://metascape.org) was used for Gene Ontology (GO) enrichment analysis.^12^ The “clusterProfiles” R package was used for Kyoto Encyclopedia of Genes and Genomes (KEGG) enrichment analysis.

### Protein–Protein Interaction Network

The STRING database (https://string-db.org/) is used to predict and construct PPI networks between the candidate genes.^13^ The DEGs were uploaded to the STRING website to obtain the PPIs. The PPI network information was imported into Cytoscape software.^14^ CytoHubba was used to identify the top 10 hub genes with high degree.

### Immune Cell Infiltration

The CIBERSORT algorithm was used to estimate the immune cell composition fractions.^15^ The algorithm was running with LM22 at 1000 permutations. Boxplots and stacked histograms were used to visualize the results of immune cell infiltration.

### Receiver Operating Characteristic Analysis

The receiver operating characteristic (ROC) analysis was performed, and area under the curve (AUC) was calculated to assess the diagnostic ability of the key genes using the “pROC” package.

### Statistical Analysis

In this study, statistical analysis was analyzed with the R version 4.2.0. Student’s *t*-tests or Wilcoxon rank-sum tests were used for analyzing the differences between the 2 groups. Correlations were performed by Pearson or Spearman’s analysis. *P* < .05 was defined as statistically significant.

## RESULTS

### Gene Expression and Enrichment Analysis Revealed Differentially Expressed Genes in Non-alcoholic Steatohepatitis–Hepatocellular Carcinoma Tissues and Their Association with the Process of Material Metabolism and Regulation of Growth

**Figure 1.**
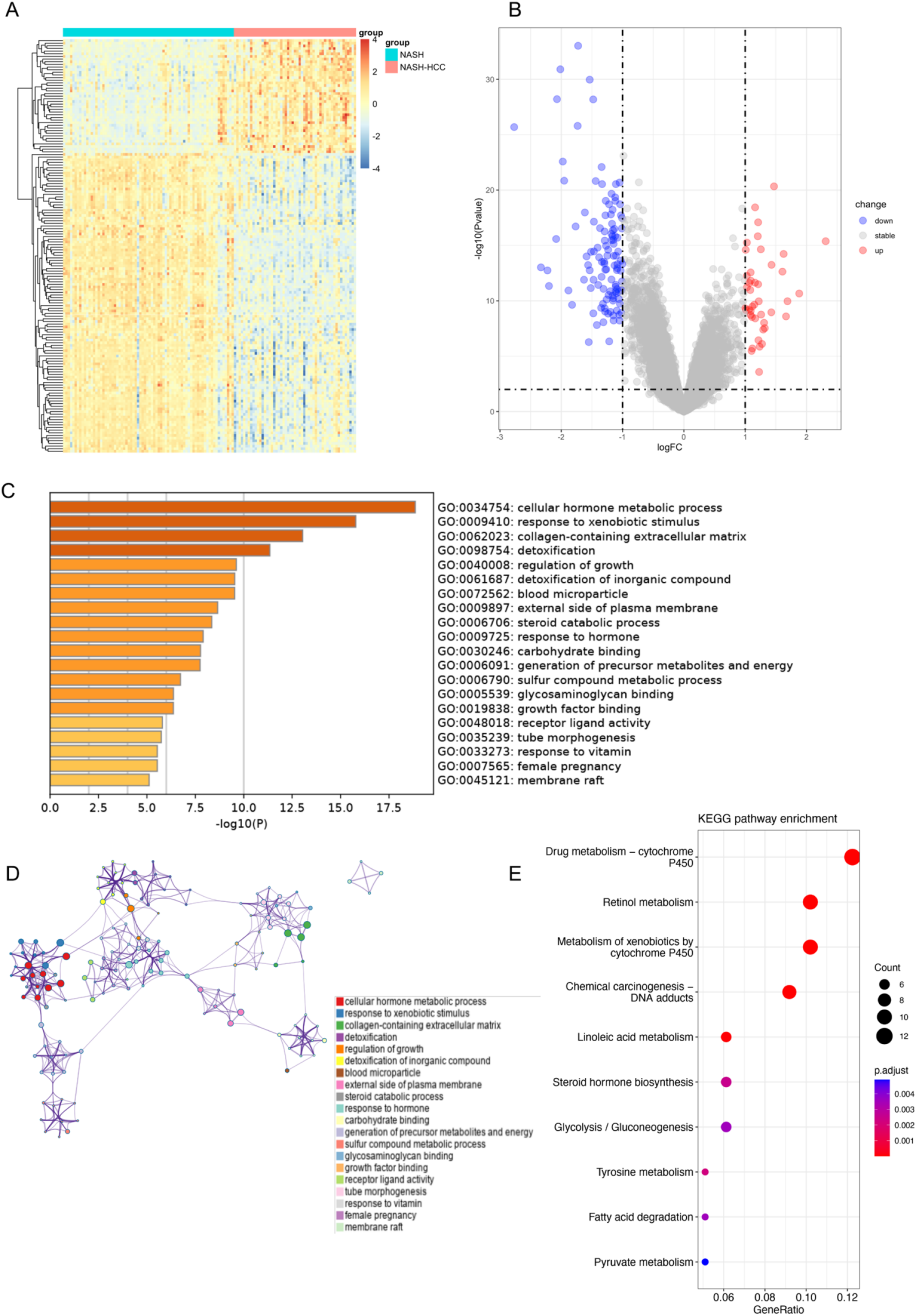
Gene expression and enrichment analysis revealed DEGs in NASH-HCC tissues and their association with the process of material metabolism and regulation of growth. (A) Heatmap of all DEGs in NASH tissues and NASH-HCC tissues. (B) Volcano map of all DEGs when comparing NASH-HCC tissues to NASH tissues. Red indicates upregulated genes, blue represents downregulated genes, and grey means genes that were not differentially expressed. (C) Heatmap of GO enrichment terms of DEGs in Metascape, colored by *P*-value. (D) Network of GO enrichment terms, colored by cluster ID. (E) The dot plot of the top 10 terms of KEGG enrichment analysis. DEG, differentially expressed genes; GO, Gene Ontology; HCC, hepatocellular carcinoma; KEGG, Kyoto Encyclopedia of Genes and Genomes; NASH, non-alcoholic steatohepatitis.

Based on 53 NASH-HCC samples and 74 NASH samples, a total of 156 DEGs from the GSE164760 dataset were acquired, including 43 upregulated and 113 downregulated DEGs in NASH-HCC tissues (Table 1). Heatmap clearly showed the landscape of genomic differences between NASH tissues and NASH-HCC tissues (Figure 1A). The volcano plot was drawn to display the distribution of DEGs between NASH-HCC tissues and NASH tissues (Figure 1B). The results of GO enrichment analysis indicated that DEGs were strongly associated with cellular hormone metabolic process, response to xenobiotic stimulus, collagen-containing extracellular matrix, detoxification, and regulation of growth (Figure 1C and 1D). The KEGG pathway demonstrated that those genes were correlated with drug metabolism–cytochrome P450, retinol metabolism, and chemical carcinogenesis–DNA adducts. (Figure 1E).

### Protein–Protein Interaction Network Established for Hub Gene Screening

**Figure 2.**
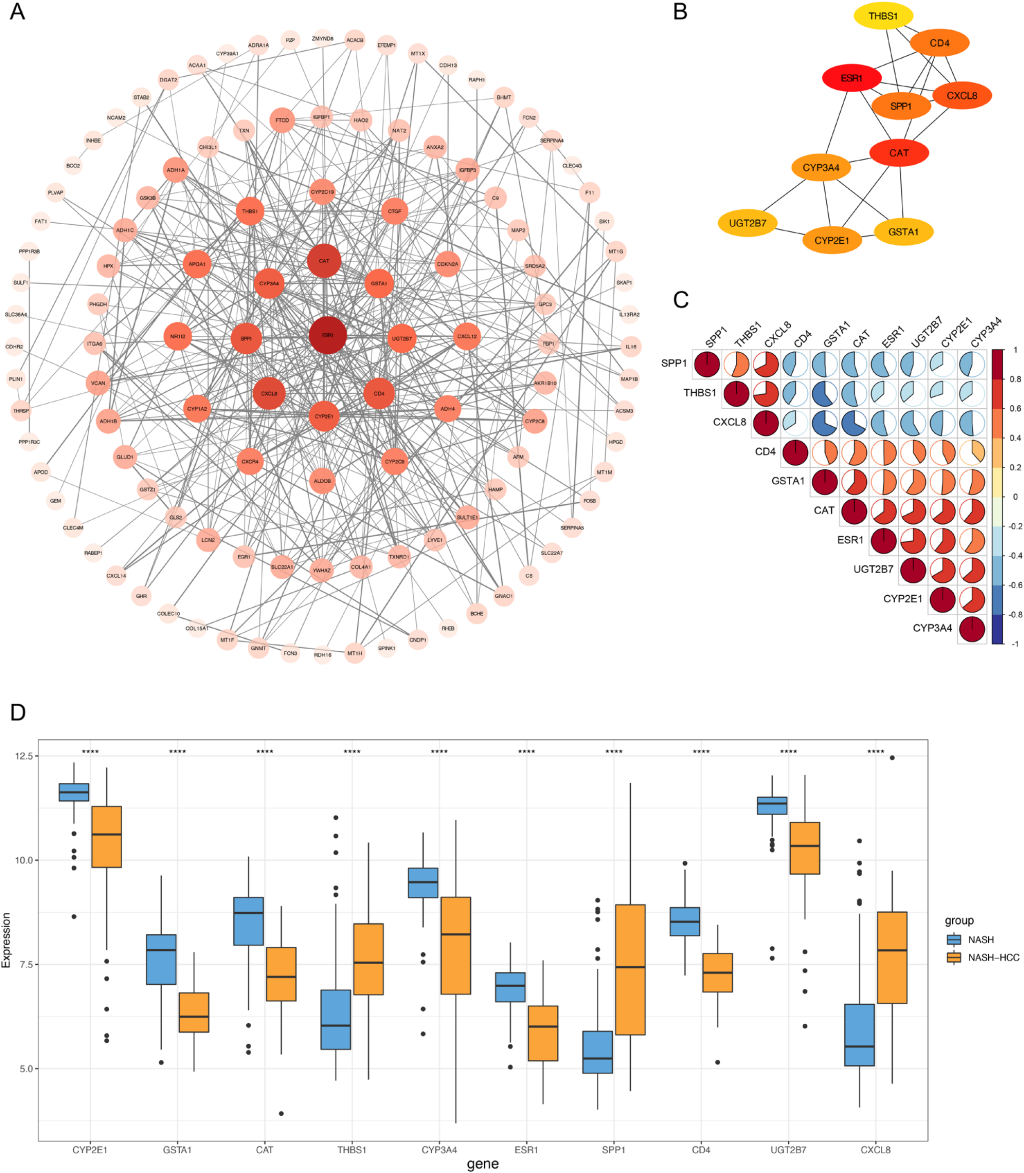
Identification of hub genes. (A) Construction of the PPI network of DEGs. The color depth and shape size of the nodes are positively correlated with degree. (B) The top 10 hub genes at protein level. (C) The correlation between the 10 hub genes. Positive correlation was marked with red and negative with blue. (D) The expression of 10 hub genes in NASH tissues and NASH-HCC tissues in GSE164760. DEG, differentially expressed genes; HCC, hepatocellular carcinoma; NASH, non-alcoholic steatohepatitis; PPI, protein–protein interaction.

The PPI network was constructed to study the relationship among those DEGs at the protein level (Figure 2A). The top 10 genes (*ESR1*, *CAT*, *CXCL8*, *CD4*, *SPP1*, *CYP2E1*, *CYP3A4*, *UGT2B7*, *GSTA1*, and *THBS1*) with the highest degree in the above network were selected as the hub genes (Figure 2B). We then investigated the correlation between these hub genes (Figure 2C). The relationship among hub genes showed that *SPP1*, *CXCL8*, and *THBS1* had a negative co-relationship with the other genes. In addition, the strongest correlation was found between *CXCL8* with *THBS1* (correlation coefficient = 0.73) and between *UGT2B7* with *ESR1* (correlation coefficient = 0.73). Then, the expression pattern of these hub genes was analyzed (Figure 2D). The expression levels of *SPP1*, *CXCL8*, and *THBS1 *were higher in NASH-HCC tissues than those in NASH tissues, while the expression levels of *ESR1*, *CAT*, *CD4*, *CYP2E1*, *CYP3A4*, *UGT2B7*, and *GSTA1* were lower in NASH-HCC tissues than those in NASH tissues.

### Immune Cell Infiltration Analysis Showed the Different Proportion of M0 Macrophages, M2 Macrophages, Plasma Cells, and CD8+T Cells Between Non-alcoholic Steatohepatitis–Hepatocellular Carcinoma Tissues and Non-alcoholic Steatohepatitis Tissues

**Figure 3.**
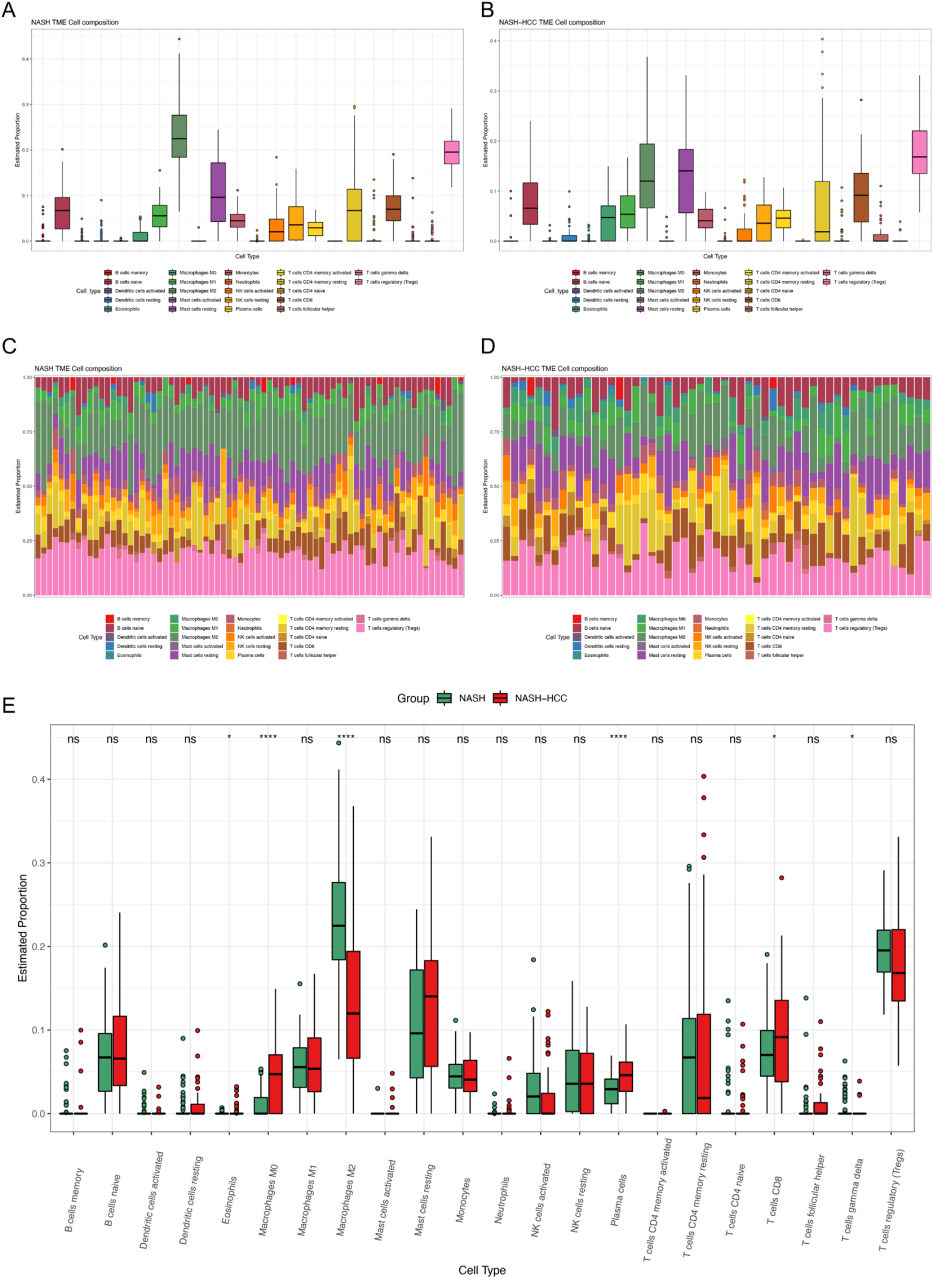
Immune cell infiltration analysis showed the different proportion of M0 macrophages, M2 macrophages, plasma cells, and CD8+T cells between NASH-HCC tissues and NASH tissues. (A) Composition of 22 immune cells in 74 NASH tissues. (B) Composition of 22 immune cells in 53 NASH-HCC tissues. (C) Fractions of 22 immune cells in 74 NASH tissues. (D) Fractions of 22 immune cells in 53 NASH-HCC tissues. (E) Comparisons of 22 immune cells between NASH tissues and NASH-HCC tissues. HCC, hepatocellular carcinoma; NASH, non-alcoholic steatohepatitis.

In order to reveal the immune microenvironment of NASH and NASH-HCC tissues, the CIBERSORT algorithm was used to analyze specific immune cell types that infiltrated into NASH and NASH-HCC tissues. The proportion of 22 immune cells in NASH and NASH-HCC tissues is shown in Figure 3A and 3B. In NASH tissues, the top 3 categories were M2 macrophages, regulatory T cells (Tregs), and resting mast cells. In NASH-HCC tissues, Tregs, resting mast cells, and M2 macrophages ranked in the top 3. Figure 3C and 3D show that the proportion of immune cells varied among samples in the NASH group and the NASH-HCC group. M2 macrophages and Tregs comprised the largest proportion of immune cell subtypes in NASH samples, and Tregs and resting mast cells occupied the highest proportion in NASH-HCC tissues. Figure 3E shows the different immune cell occupancies in NASH samples and NASH-HCC samples. M0 macrophages, plasma cells, and CD8+T cells were higher in NASH-HCC tissues, while M2 macrophages were higher in NASH tissues.

**Figure 4.**
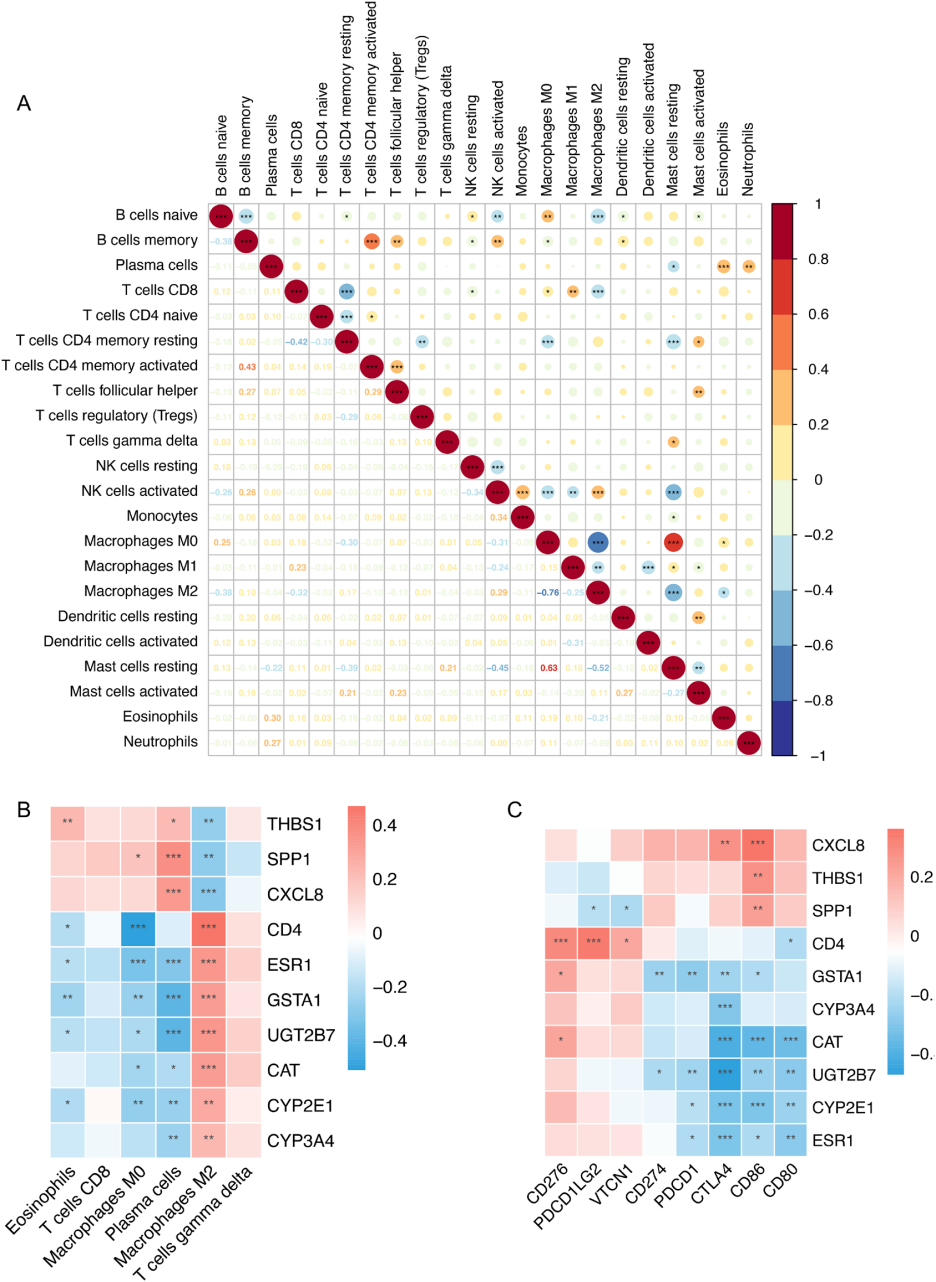
Correlation of hub genes and immune cells. (A) The correlation of 22 immune cells. Red represents positive correlation and blue represents negative correlation. Lower panel was Pearson’s correlation coefficient. In the upper panel, the size of the circle is positively correlated with the correlation coefficient. (B) The correlation between each hub gene and changed immune cells. Red, positive; blue, negative. (C) Correlation between the 10 hub genes and 8 immune checkpoint genes.

Then, the correlation matrix was drawn to visualize the interaction of 22 immune cells. As shown in Figure 4A, by further analyzing the CIBERSORT scores, M2 macrophages had the highest negative relationship with M0 macrophages, while resting mast cells had the highest positive correlation with M0 macrophages. Furthermore, we explored the relationship between the 10 hub genes and the significantly changed immune cells. As shown in Figure 4B, M2 macrophages showed a consistent correlation among the 10 hub genes. Moreover, we analyzed the relationship between the 10 hub genes and 8 immune checkpoint genes, including “*CD274*,” “*CTLA4*,” “*PDCD1*,” “*PDCD1LG2*,” “*CD86*,” “*CD80*,” “*CD276*,” and “*VTCN1*” (Figure 4C). *UGT2B7* showed the highest negative correlation with *CTLA4* (*R* = –0.47).

### Hub Gene Verification and Diagnostic Ability Assessment Indicated That *CD4*, *UGT2B7*, and *CYP3A4* may be the Potential Diagnostic Biomarkers of Non-alcoholic Fatty Liver Disease–Hepatocellular Carcinoma

**Figure 5.**
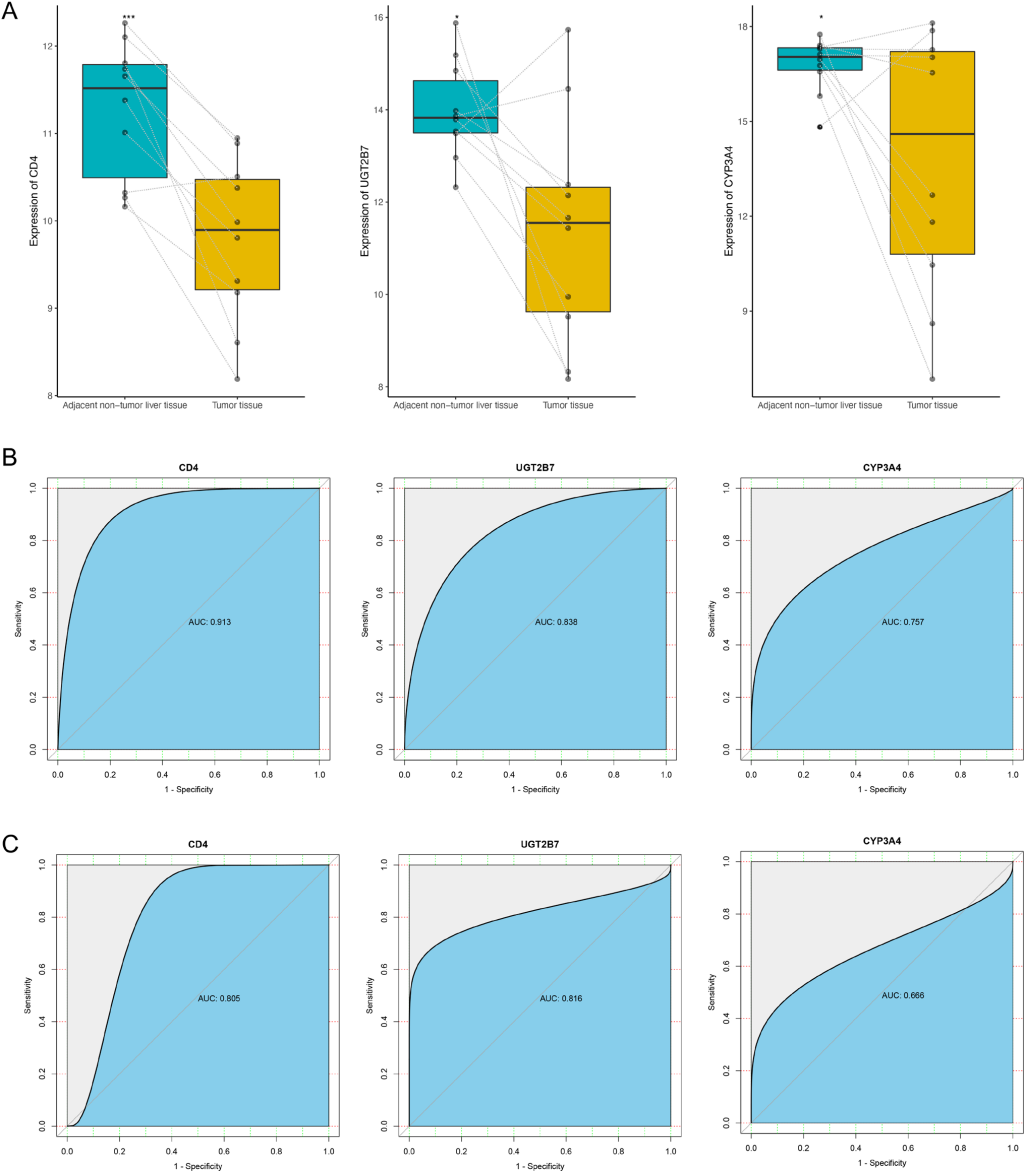
Hub gene verification and ROC analysis indicated that *CD4*, *UGT2B7*, and *CYP3A4* may be the potential diagnostic biomarkers of NAFLD-HCC. (A) The differential expression of *CD4*, *UGT2B7*, and *CYP3A4* in paired tumor tissues and adjacent tissues of GSE164441. (B) ROC analysis of *CD4*, *UGT2B7*, and *CYP3A4* in GSE164760. (C) ROC analysis of *CD4*, *UGT2B7*, and *CYP3A4* in GSE164441. HCC, hepatocellular carcinoma; NAFLD, non-alcoholic fatty liver disease; ROC, receiver operating characteristic.

To verify the expression of the 10 hub genes in NAFLD-HCC, we downloaded the dataset GSE164441 (RNA-Seq). As it is shown in Figure 5A, of the 10 hub genes, only the expression levels of *CD4*, *UGT2B7*, and *CYP3A4* were significantly different in the dataset GSE164441, and all the 3 genes were downregulated in tumor tissues. Then, the ROC analysis was performed to evaluate the sensitivity and specificity of *CD4*, *UGT2B7*, and *CYP3A4* for the diagnosis of NAFLD-related HCC. In the dataset GSE164760, the AUC of *CD4*, *UGT2B7*, and *CYP3A4* was 0.913, 0.838, and 0.757, respectively (Figure 5B). In the verification dataset GSE164441, the AUC values of *CD4*, *UGT2B7*, and *CYP3A4* were 0.805, 0.816, and 0.666, respectively (Figure 5C). The results indicated that *CD4*, *UGT2B7*, and *CYP3A4* may be potential diagnostic biomarkers of NAFLD-HCC.

## DISCUSSION

In the study, we obtained 156 DEGs between NASH tissues and NASH-HCC tissues and discovered that those genes were related to the process of material metabolism and regulation of growth. Subsequently, 10 hub genes were selected via the PPI network. The results of immune infiltration analysis showed that M2 macrophages, Tregs, and resting mast cells comprised the largest proportion of immune cell subtypes both in NASH samples and in NASH-HCC samples. The proportion of M0 macrophages, plasma cells, and CD8+T cells in NASH-HCC tissues is significantly increased compared with NASH tissues; in contrast, the proportion of M2 macrophages in NASH-HCC tissues is significantly decreased. Then, we verified hub genes in another dataset, and only the expression of *CD4*, *UGT2B7*, and *CYP3A4* was significantly different, which may be due to the differences in detection methods and samples. Finally, we found the 3 genes could serve as the prospective markers for NAFLD-to-HCC transition via ROC analysis.

Tumor immune microenvironment plays a significant role in the transition from NAFLD to HCC. Immunotherapy such as immune checkpoint inhibitors holds great promise for advanced HCC; however, recent data suggested that patients with NAFLD-associated HCC are less sensitive to conventional immune checkpoint inhibition due to the altered immune components.^16-18^ Studies have reported that the activation of intrahepatic CD8+T cells promotes the NASH-to-HCC transition.^19,20^ Pfister et al^7^ revealed that aberrantly activated CD8+PD1+ T cells in the TME involve in NASH-induced liver injury and progression of NASH to HCC, which may limit the response to immunotherapy. Macrophages include 3 subtypes (M0, M1, and M2), and each subtype has different functions in tumorigenesis and development. Previous studies have reported that M0 macrophage enrichment is associated with poor prognosis and sorafenib response in HCC.^21,22^ M2 macrophages serve a significant role in facilitating cancer initiation, angiogenesis of tumor stroma, and immunosuppression.^23-25^ In our study, although the proportion of M2 macrophages in NASH-HCC tissues was lower than that in NASH tissues, its proportion in the NASH-HCC tissues was still significantly higher than that of other immune cell types.

*CD4* molecule plays an important role in the development, differentiation, activation, and antigen recognition of T cells. CD4+T cells exert an important role in tumor immune surveillance. The absolute number of CD4+T cells is decreased in the inflamed liver and NAFLD-promoted HCC.^20,26^ A study suggested that selective loss of CD4+T lymphocytes in NAFLD promotes hepatocarcinogenesis.^27^ It is also reported that CD4+T cells inhibit the initiation of HCC and contribute to tumor regression.^28,29^

*UGT2B7* (UDP glucuronosyltransferase family 2 member B7) is a member of the UGT2B family, is mainly expressed in the liver, and is involved in biotransformation and detoxification. The altered expression of *UGT2B7* may affect the toxicity and efficacy of drugs.^30^ It was reported that the expression of *UGT2B7* was downregulated in HCC, and the impaired function was closely related to the hepatocarcinogenesis.^31^ In addition, *UGT2B7* was strongly associated with microvascular invasion in HCC.^32^

*CYP3A4* (cytochrome P450 family 3 subfamily A member 4), mainly expressed in the liver and intestine, is involved in the majority of drug metabolism, participating in the metabolism of some pre-carcinogens.^33,34^ The downregulation of *CYP3A4* might influence the metabolism of some pre-carcinogens, which might be associated with carcinogens. *CYP3A4* was reported as a potential diagnosis and prognosis biomarker for HCC.^35-37^

However, this study has some limitations that need to be focused in future work. First, we only used 1 gene expression profile for differential gene analysis, which may result in selection bias. Second, the study was based on the bioinformatics analysis of the GEO dataset and experiments should be conducted next to validate the results. Besides, we did not analyze the clinicopathological parameters due to the lack of clinical information.

In summary, by bioinformatics analysis, we identified and verified the hub genes associated with NAFLD-to-HCC transition and found that *CD4*, *UGT2B7*, and *CYP3A4* may serve as the potential biomarkers of NAFLD-HCC. This recognition may provide new insight into the understanding of the pathogenesis of NAFLD-to-HCC transition.

## Figures and Tables

**Table 1.** DEGs in NASH-HCC Samples Compared with NASH Samples

DEGs	Gene Name
Upregulated	*IGFBP1, **SPP1**, CXCR4, **CXCL8,** CDH13, SPINK1, CDKN2A, AKR1B10, LCN2, CHI3L1, EGR1, EFEMP1, APOD, ANXA2P2, C1orf198, GEM, KDM5D, VCAN, TXN, PLVAP, SULF1, ZIC2, SIK1, CAP2, FAT1, SNORA71D, FOSB, MUC13, ANXA2, **THBS1**, COL4A1, ITGA6, YWHAZ, CCN2, MAP2, TXNRD1, SPRY1, COL15A1, OLFML2B, STC1, MAP1B, GPC3, NSMCE4A*
Downregulated	*CLEC4G, FCN2, VIPR1, CLEC4M, STAB2, FCN3, CRHBP,*
	*CXCL14, SLC19A3, CD5L, GLYAT, ACACB, GNAO1,*
	*IGFBP3, DNASE1L3, ACAA1, IL13RA2, FAM13A, GHR, **CD4**,*
	*NR1I2, DGAT2, C1RL-AS1, COLEC10, CYP39A1, OBSL1,*
	*CLTRN, ADRA1A, CNDP1, NAT2, SERPINA4, GSTZ1,*
	*BCO2, SKAP1, SULT1E1, LIFR, F11, RAPH1, MT1F, FTCD,*
	*KCNN2, LYVE1, GLUD1, RABEP1, AFM, FXYD1, ACSM3,*
	*CDHR2, THRSP, ZG16, GASK1A, CXCL12, CYP2C9,*
	*CYP2C8, CYP2C19, PPP1R1A, HSD17B13, **GSTA1**, AGBL2,*
	*PLAC8, ADH4, APOA1, MT1H, SRD5A2, ADH1A, GBA3,*
	*RDH16, CYP1A2, MT1G, PHGDH, PZP, PBLD, BCHE,*
	*BHMT, **ESR1,** HPX, PPP1R3C, HAMP, GNMT, ATF5, **CAT**,*
	*SLC22A7, FYB2, MT1JP, INHBE, GSK3B, SERPINA5, PLIN1,*
	*TRO, HAO2, PPP1R3B, SLC38A4, RHEB, IL16, ANXA10,*
	*ALDOB, MT1M, SLC22A1, **CYP2E1**, **UGT2B7**, GLS2, HPGD,*
	*FBP1, **CYP3A4**, ADH1C, C6, PXMP2, MT1X, NCAM2,*
	*ADH1B, C9, CFHR3, XIST*

A total of 43 upregulated DEGs and 113 downregulated DEGs were identified in NASH-HCC tissues, compared with NASH tissues. The hub genes are shown in boldface.

DEGs, differentially expressed genes; HCC, hepatocellular carcinoma; NASH, non-alcoholic steatohepatitis.
